# Risk factors and prevention strategy for patient dissatisfaction after transforaminal lumbar interbody fusion: a single-center retrospective study

**DOI:** 10.3389/fsurg.2025.1545591

**Published:** 2025-02-11

**Authors:** Lixin Yang, Zhao Guo, Yuhao Qiao, Jichao Guo, Jiaqi Li, Wei Wang

**Affiliations:** ^1^ Orthopedics Department, Hebei Medical University Third Hospital, Shijiazhuang, Hebei, China; ^2^ Orthopedics Department, Affiliated Hospital of Hebei University, Baoding, Hebei, China

**Keywords:** transforaminal lumbar interbody fusion, lumbar disc herniation, dissatisfaction, risk factors, prevention strategy

## Abstract

**Background:**

For patients who received transforaminal lumbar interbody fusion (TLIF) treatment for lumbar disc herniation, most of them can achieve good results, but there were still some patients who were not satisfied with the surgical results. The purpose of this study is to explore the factors that contribute to patient dissatisfaction after TLIF.

**Methods:**

From March 2018–December 2021, patients with lumbar disc herniation who received TLIF treatment were included in this study. Clinical data from preoperative and postoperative 2-year follow-up were analyzed. Associations between clinical variables and function of postoperative were examined in univariate and multivariate analysis.

**Result:**

Of all the 625 patients, including 296 (47.4%) male patients and 329 (52.6%) female patients. According to patient satisfaction index (PSI), patients were divided into two groups, 529 patients in satisfied group showing 1 or 2 stage in PSI and 96 patients in dissatisfied group showing 3 or 4 stage in PSI. Univariate analysis showed that body mass index (BMI), preoperative pain time, postoperative visual analog scale (VAS)-back, intraoperative bleeding volume, postoperative rehabilitation training, feel depression and symptom recurrence were related with patient's level of satisfaction 2 years after discectomy. When incorporated into a multivariate logistic regression analysis, it was found that BMI, preoperative pain duration, postoperative rehabilitation training, feel depression and symptom recurrence were individually correlated with patient dissatisfaction 2 years after discectomy.

**Conclusion:**

This study indicates that the factors that leading to postoperative dissatisfaction of patients include obesity, preoperative pain duration lasting no less than 6 months, feel depression and symptom recurrence. While postoperative rehabilitation training for three months is a protective factor.

## Introduction

Lumbar disc herniation is a common spinal surgical disease, especially in elderly population, with low back pain and sciatica being its main clinical symptoms (1, 2). Although it usually responds favorably to conservative treatment, surgical decompression is necessary in a small percentage of them. For patients with unilateral disc herniation, microscopic surgery is a common choice to minimize the damage (3, 4), but microscopic or endoscopic minimally invasive approaches may not be suitable for all cases. Studies have suggested that minimally invasive techniques may not be effective enough to achieve satisfactory outcomes in Carragee type II and IV hernias, which are more prone to recurrent disc herniation and segmental instability (5, 6). Clinical evidence shows that decompression surgery such as lumbar discectomy and vertebral fusion can improve low back pain and sciatica (7, 8). For patients with unilateral disc herniation, the less trauma surgery such as transforaminal lumbar interbody fusion (TLIF) can also improve both lower back pain and sciatica and most patients can achieve good results, but there are still some patients who are not satisfied with the surgical results (9).

The purpose of performing lumbar surgery is to alleviate the pain of the patients and achieve satisfactory therapeutic effects. Satisfaction or dissatisfaction are both subjective feelings experienced by patients. However, the factors that lead to dissatisfaction among patients undergoing transforaminal lumbar interbody fusion surgery are not yet known. In view of this, the objective of this study is to investigate the factors contributing to patients' dissatisfaction following TLIF surgery, in order to offer valuable insights to clinicians.

## Materials and methods

### Patients

We conducted a retrospective analysis of 625 patients who received TLIF from March 2018 to December 2021. Informed consent was obtained from each patient, and the study was approved by the ethics review board of our hospital and acquired a unique identification number for research registration (the research registration number is 20241111).

The inclusion criteria consisted of patients who: (1) were aged 18 years or older, (2) presence of mechanical back and unilateral radicular leg pain caused by lumbar disc herniation, (3) failure of conservative treatment to alleviate the radicular pain, (4) confirmation of lumbar disc herniation through magnetic resonance imaging (MRI) findings, and (5) follow up to study endpoint. Exclusion criteria were: (1) Other severe spinal diseases such as spinal tumors, spinal tuberculosis, and pyogenic spinal infections, (2) spinal mechanical instability, such as lumbar spondylolisthesis or segmental instability, (3) unwillingness to participate in the study, and (4) lumbar spine surgery history.

### Surgical technique

After general anesthesia, the patient is placed in a prone position with the abdomen suspended to reduce intra-abdominal pressure, thereby reducing bleeding from the venous plexus in the spinal canal during decompression operations. A midline incision on the lower back should be made, cutting through the skin and subcutaneous tissue. Then, the paraspinal muscles should be carefully removed from under the periosteum to reveal the vertebral plate and facet joints. Pedicle screws were placed, and then the lower articular process of one side of the upper vertebral body and the upper articular process of the lower vertebral body was bitten off by the vertebral plate clamp. When removing the ligamentum flavum, the dural sac is protected by the nerve peeler to prevent tearing the dura mater. The deep ligamentum flavum was removed and the lateral recess was decompressed. Then the hypertrophic ligamentum flavum and residual joint capsule were removed from the intervertebral foramen and lateral recess.

Expose the intervertebral disc from the intervertebral foramen area. Use a sharp knife to cut through the fibrous ring on the surface of the intervertebral disc. Use nucleus pulposus forceps to remove degenerated nucleus pulposus tissue from the intervertebral disc. Scrape off the upper and lower cartilage endplates and determine the type of intervertebral fusion cage. Wash the intervertebral space with sterile physiological saline, implant autologous bone, allogeneic bone fragments, and fusion cage and ensure the presence of bone tissue around the fusion cage. Place the appropriate length of the bowl rod and bending, and place the screw tail cap. Cover the surface of the dural sac with gelatin sponge. Place drainage tube and suture the layers.

### Clinical indexes

This study includes the collection of clinical indicators from the cases and follow-up results at 2 years after surgery. These include age, gender, osteoporosis, body mass index (BMI), painful limbs, hypertension, diabetes, occupation, lesion segment (multi segment, high-level segment), preoperative pain time, operation time, intraoperative blood loss, postoperative wound drainage, wound infection, deep vein thrombosis (DVT), preoperative visual analog scale (VAS)-back, postoperative VAS-back, postoperative rehabilitation training, complication, feel depression 2 years after discectomy, symptom recurrence and level of satisfaction.

Osteoporosis was diagnosed with the aid of x-ray or computerized tomography (CT) findings. In continuous variables, except for age and BMI, all other variables are defined by median as their high and low values. Obesity was defined as having a BMI of no less than 28. Advanced age is defined as the patient's age not less than 60 years old. Patients were divided into mental workers and manual workers according to their occupation. The patients, who undertook a small amount of manual labor and worked mainly indoor, were regarded as mental workers. The patients, who undertook a large amount of manual labor and worked mainly outdoor, were regarded as manual workers. The level of patient satisfaction with the treatment outcome was determined using the patient satisfaction index (PSI) (10). A PSI of 1 or 2 is considered satisfactory, while a PSI of 3 or 4 is considered unsatisfactory. DVT records did not include cases of lower extremity intermuscular thrombosis because this type of thrombosis was classified as a peripheral variant of DVT and essentially limited to the venous plexus of soleus and gastrocnemius. Studies have shown that lower extremity venous intermuscular thrombosis has almost no impact on patients (11). Postoperative rehabilitation training starts on the second day after surgery and lasts until three months after surgery and it is mainly for lumbar core muscle training. The Zung Self-Rating Depression Scale (ZSRDS) (12), a 20-item self-administrated questionnaire, was used to measure the severity of depressive symptoms. ZSRDS consists of 10 express negative experience and 10 express positive experience. The ZSRDS scores on the test range from 25 through 100. An ZSRDS score = 50 suggests clinically significant symptoms with the following three levels of severity ratings: score 25–49 response normal; score 50–59 response mild to moderate; score 60–69 response moderate to severe; and score no less than 70 responses severe (13, 14). Symptom recurrence is a result discussed by the group of expert and manifests as lower back pain and sciatica caused by lumbar disc herniation either on the opposite side of the initial treatment site or in adjacent segments.

### Follow up and end point

Follow up of patients after discharge is necessary. Generally, patients are scheduled for regular follow-up examinations at one month, three months, six months, one year and two years after surgery. However, it is important to note that if patients experience sudden situations such as significant back pain and lower limb neuralgia, they can come for diagnosis at any time. This study would have two endpoints. One is that during a 2-year period, the patient experienced severe back pain and lower limb neuralgia again and after being diagnosed with symptom recurrence through MRI examination, the time and VAS-back were recorded. The other is 2 years after surgery, at this point, all patients except those who have already completed the study would be evaluated.

### Statistics

This study is based on binary logistic regression analysis to calculate the minimum sample size required. In this study, stepwise binary logistic regression was applied to eliminate confounding factors, and patient dissatisfaction level was used as the dependent variable for comparative analysis. The chi square test was used for univariate analysis, and variables with *p* ≤ 0.05 were separately subjected to binary logistic regression analysis to exclude confounding variables with *p* > 0.05. Then, the remaining variables were included in the binary logistic regression model with dissatisfaction level as the dependent variable. For each variable, we calculated the odds ratio (OR) of its 95% confidence interval (CI). In the chi-square test and multivariate logistic regression model, except for age and BMI, all the other continuous variables were defined by the median. All statistical analyses were done using SPSS software version 27.0 (SPSS, Inc., Chicago, IL, USA).

## Result

### General information

According to the sample size calculation formula, the minimum sample size required for this study is 323, which is much smaller than the actual sample size in this study. A total of 660 patients were included in the study. Of these, 24 patients with spinal mechanical instability and 11 who refused the second evaluation were excluded. Finally, 625 patients who met the inclusion criteria from March 2018 to December 2021 were included in this study. Among them, there were 296 male patients (47.4%) and 329 female patients (52.6%). The average age and BMI were 54.9 years old and 26.48 kg/m^2^, respectively. According to PSI, patients are divided into two groups, 529 patients (84.6%) in satisfied group showing 1 or 2 stage in PSI and 96 patients (15.4%) in dissatisfied group showing 3 or 4 stage in PSI (Table 1).

**Table 1:** Patient satisfaction index (PSI).

PSI	Patient Responses	The level of satisfaction	Patients(*n* = 625)
1	Surgery met my expectations	Satisfied	529
2	Surgery improved my condition enough so that I would go through it again for the same outcome		
3	Surgery helped me but I would not go through it again for the same outcome	Dissatisfied	96
4	I am the same or worse compared to before surgery		

PSI, patient satisfaction index.

### Univariate analysis of factors related to postoperative patient dissatisfaction

At baseline, there was no difference between the two groups in gender, age, occupation, hypertension, diabetes, painful limbs and preoperative VAS-back. Compared to satisfied patients, the dissatisfied patients had longer preoperative pain time and higher BMI (Table 2). For the collected clinical information, there was no difference between the two groups in terms of surgical segment (including high and multiple segments), surgical time, wound drainage volume, and postoperative thrombosis. Compared to satisfied patients, dissatisfied patients had higher postoperative VAS-back, intraoperative bleeding volume, incidence of depression and symptom recurrence rate (Table 3).

**Table 2:** Basic information of 625 patients.

Parameters	Satisfied (*n* = 529)	Dissatisfied (*n* = 96)	*X^2^*	*P*
Gender				
Male	259	37	3.538	0.60
Female	270	59		
Age (average)	55.0	54	1.207	0.272
Occupation				
Mental workers	135	20	0.957	0.328
Manual workers	394	76		
BMI				
≥28	183	58	22.870	<0.001
<28	346	38		
Hypertension				
Yes	146	28	0.099	0.753
No	383	68		
Diabetes				
Yes	57	9	0.169	0.681
No	472	87		
Painful limbs				
Left	271	58	2.751	0.097
Right	258	38		
Preoperative pain time (month) (average)	5.4	7.3	21.598	<0.001
Preoperative VAS-back (average)	5.8	5.9	3.616	0.057

*X^2^* means chi-square test; *P* means *P* value.

**Table 3:** Surgical related information and 2-year follow-up after surgery of 625 patients.

Parameters	Satisfied (*n* = 529)	Dissatisfied (*n* = 96)	*X^2^*	*P*
Surgical segment				
Multi segment	142	31	1.025	0.272
High-level segment	70	18	2.045	0.153
Surgical time (minute) (average)	140	145	0.936	0.333
Intraoperative bleeding volume (ml) (average)	579	625	4.422	0.035
Wound drainage volume (ml) (average)	357	367	0.116	0.733
Postoperative thrombosis				
Yes	58	9	0.214	0.643
No	471	87		
Rehabilitation training				
Yes	243	28	9.578	0.002
No	283	68		
Symptom recurrence				
Yes	9	75	407.931	<0.001
No	520	21		
Postoperative VAS-back (average)	1.1	4.7	142.188	<0.001
Feel depression				
Yes	12	60	289.200	<0.001
No	517	36		

*X^2^* means chi-square test; *P* means *P* value.

### Handling of confounding factors

Perform binary logistic regression analysis separately with postoperative dissatisfaction level as the dependent variable for the following factors: BMI, preoperative pain duration, intraoperative bleeding volume, postoperative VAS-back, feel depression, postoperative rehabilitation training and symptom recurrence. When comparing the above factors with postoperative dissatisfaction levels, no confounding factors were found, and they all had statistical significance. Table 4 showed detailed data for excluding confounding factors.

**Table 4:** Individual logistic regression analysis excludes confounding factors.

	B value	Se value	Wald value	*P* value	Or value	95% CI
Preoperative pain duration	1.116	0.248	20.188	<0.001	3.052	1.876–4.965
Intraoperative bleeding volume	0.495	0.237	4.364	0.037	1.641	1.031–2.612
BMI	1.060	0.228	21.637	<0.001	2.886	1.846–4.510
Symptom recurrence	5.330	0.417	163.256	<0.001	206.349	91.107–467.362
Postoperative VAS-back	5.279	1.027	26.421	<0.001	196.114	26.204–1,467.766
Rehabilitation training	0.735	0.241	9.301	0.002	2.085	1.300–3.344
Feel depression	4.274	0.360	140.825	<0.001	71.806	35.448–145.453

95% CI, 95% confidence interval.

### Factors of patient dissatisfaction after discectomy identified by multivariate analysis

The following variables were included as input into the multivariate model: BMI, preoperative pain duration, intraoperative bleeding volume, postoperative VAS-back, feel depression, rehabilitation training and symptom recurrence. When included in a multivariate logistic regression model, BMI, preoperative pain duration, rehabilitation training, feel depression and symptom recurrence were independently associated with patient dissatisfaction 2 years after discectomy (Table 5).

**Table 5:** Factors of patient dissatisfaction after discectomy identified by multivariate analysis.

	B value	Se value	Wald value	*P* value	Or value	95% CI
Preoperative pain duration	1.061	0.458	5.378	0.020	2.890	1.179∼7.084
Intraoperative bleeding volume	0.009	0.439	0.00	0.984	1.009	0.427∼2.383
BMI	1.061	0.449	5.590	0.018	2.889	1.199∼6.963
Symptom recurrence	4.786	0.569	70.747	<0.001	119.808	39.279∼365.438
Postoperative VAS-back	−0.741	1.254	0.349	0.555	0.477	0.041∼5.569
Rehabilitation training	0.970	0.482	4.056	0.044	2.638	1.026∼6.781
Feel depression	2.859	0.597	22.931	<0.001	17.439	5.412∼56.192

95% CI, 95% confidence interval.

## Discussion

In this study, a total of 625 patients with lumbar disc herniation treated with TLIF were investigated. Most patients were satisfied with the results of discectomy and fusion surgery, and would undergo surgery again. However, there are still 96 patients (15.4%) who are dissatisfied, and there is sufficient evidence to suggest that we should identify preoperative and postoperative factors that affect patients' level of satisfaction, in order to maximize the therapeutic benefits for patients. This study shows that obesity, preoperative pain lasting more than three months, feel depression and recurrence of symptoms are four independent factors affecting patients' dissatisfaction. While postoperative rehabilitation training for three months is a protective factor. Therefore, in the treatment of lumbar disc herniation with TLIF, it is important to pay close attention to these factors to improve the recovery effect.

This study shows that obesity is considered to be an independent factor for dissatisfaction in patients undergoing TLIF surgery. Obesity is often a negative factor for patients after surgery, which may be related to the following two aspects. First of all, compared with normal weight controls, obese patients experience an increase in intervertebral disc pressure during most daily activities, leading to accelerated intervertebral disc degeneration (15, 16), and studies have shown than obesity is associated with adjacent segment degeneration after lumbar fusion for degenerative lumbar disease (17). The degeneration of adjacent segments will lead to a series of new problems, such as symptom recurrence (18), thereby reducing the psychological expectations of obese patients. Moreover, obesity frequently hampers the overall activity levels of postoperative patients, leading to decreased mobility and potentially diminishing the surgical outcome. However, obesity should not be considered a contraindication for patients undergoing surgery, and for the improvement of back pain in patients, obesity is comparable to non-obese patients. Therefore, preoperative conversations with obese patients are necessary.

Previous studies have not provided sufficient evidence to demonstrate the relationship between duration of preoperative symptoms and postoperative satisfaction for patients undergoing TLIF surgery. In this study, we found that preoperative symptom duration lasting no less than six months was an independent risk factor for postoperative dissatisfaction. As the preoperative symptom duration increased, the patient 's dissatisfaction increased. On the one hand, the longer duration of preoperative symptoms is related to the longer duration of nerve root compression, which may lead to irreversible damage to the nerve root (19). On the other hand, after the first symptoms appear, patients usually choose conservative treatment. In China, most patients consider the cost issue and surgery is usually considered as the last option for treatment, with high expectations. However, after undergoing surgical treatment, some patients did not achieve the expected surgical outcome. It is still uncertain whether a preoperative symptom duration of 6 months can be used as a standard timeline for surgical efficacy. Previous studies have shown that patients with preoperative symptom duration less than three months achieved better results one year after surgery, and predicted that the rate of re-operation increased with the prolongation of preoperative symptom duration (20). Other studies have also reported that patients with preoperative symptoms lasting longer than one year had adverse outcomes, whether it was in the early or middle and late postoperative period (21). Patients who undergo early surgery are more likely to benefit more than patients with prolonged symptoms.

The ZSRDS is a 20-item questionnaire with well-established reliability and validity (22). Postoperative depression measured by the ZSRDS is another factor of dissatisfaction after TLIF. The association between preoperative depression and postoperative outcomes was demonstrated in previous studies, that is, preoperative depression reduces patients' level of satisfaction (23, 24). However, we found that postoperative depression also affects patients' level of satisfaction in this study. On the one hand, compared to preoperative depression, which is influenced by prolonged pain and high surgical costs, postoperative depression largely depends on the effectiveness of the surgery, indirectly reflecting the impact of psychological states such as feeling depressed on postoperative satisfaction. On the other hand, patients with depression may be more sensitive to postoperative pain, which may further reduce postoperative satisfaction (25). For patients undergoing TLIF, changes in postoperative depression have a more significant impact on satisfaction than preoperative depression (25). It is necessary to understand the expectations of these patients and fully provide them with emotional support and physical therapy to improve their overall satisfaction. Therefore, it is necessary to conduct preoperative visits by anesthesiologists and strengthening preoperative education, anxiety and depression can be detected early and intervened in a timely manner, which can reduce postoperative pain and cognitive dysfunction. The clinical doctors should inform patients of the surgical effect to avoid unrealistic expectations and alleviate surgical anxiety. Postoperative appropriate medication should be used to stabilize the patient's emotions and eliminate adverse reactions such as tension, fear, and anxiety caused by various surgeries. During postoperative follow-up, appropriate communication should be given to the patient. If symptoms are found, a psychologist should be consulted and medication should be used for treatment.

Another factor that affects postoperative dissatisfaction of patients is symptom recurrence, which often requires secondary surgery, which brings additional physical and psychological trauma to patients. It is reported that the incidence of recurrent lumbar disc herniation is 5%–15% (26, 27). This study showed that 84 (13.4%) of the 625 patients who received TLIF for lumbar disc herniation symptom recurrence. Among these recurrent patients, the majority were not satisfied with the results two years after surgery, while 9 patients (10.7%) still showed their satisfaction. We found that among the 9 patients who were satisfied, two patients had obvious obesity, five patients had long preoperative pain, and two patients had high preoperative VAS-back. Although the symptoms recurred after surgery, the pain was still relieved. The above patients were satisfied with the preoperative full communication and underwent secondary surgery. Unfortunately, this study did not conduct further follow-up. The recurrence of symptoms in patients treated with TLIF for lumbar disc herniation can occur either on the opposite side of the initial treatment site or in adjacent segments. Symptoms recurrence is the most common cause of unsatisfactory surgical treatment in patients with lumbar disc herniation (26, 28). Good preoperative communication can enable patients to fully understand the evolution of the disease, reduce the fear of the disease, and increase the level of postoperative satisfaction.

Postoperative rehabilitation exercise is the last factor affecting patient satisfaction in this study. Rehabilitation exercise is a protective factor, and patients who undergo rehabilitation exercise after surgery are more likely to achieve satisfactory surgical results. Postoperative rehabilitation training can effectively relieve pain and improve quality of life, and early rehabilitation training could enhance results in terms of pain and disability without an enhanced risk of complications (29, 30). However, in practical clinical work, rehabilitation exercise is often overlooked, even though both clinical doctors and patients know the effectiveness of rehabilitation exercise. In this study, we will inform patients in detail about the importance of postoperative rehabilitation exercise after surgery, but only 43.36% (271/625) patients persisted in exercising every day until 3 months after surgery. The effect of rehabilitation exercise is slow and firm (31), and rehabilitation training can improve muscle and tissue tolerance, avoiding slow recovery caused by muscle atrophy (29). Therefore, postoperative functional exercise is needed to strengthen the waist muscles, alleviate patient pain, and improve patient satisfaction.

There are several limitations in our research. Firstly, satisfaction is the subjective reaction of patients to clinical results, which is related with many variables and may result in selection bias. Secondly, this study is a retrospective single center study, which is still a valuable research method despite limitations such as limited the ability to establish causal relationships, recall bias, and limited generalizability of results. This article can provide clues and hypotheses for future research through reasonable design and rigorous analysis. Meanwhile, future research should consider conducting a multicenter prospective study with a larger sample size to validate our results. Finally, considering that precise osseointegration of the vertebral body and ideal recovery period of neurological function can be achieved 2 years after surgery, which can lead an accurate result. Therefore, this study only recorded detailed data of patients 2 years after surgery, and due to the limitations of retrospective analysis, detailed data of patients who underwent secondary surgery 3 months, 6 months, 1 year, and 2 years after surgery were not recorded in a timely manner.

## Conclusion

This study indicates that 84.6% of patients are satisfied with TLIF treatment for lumbar disc herniation, but there are still some patients who are dissatisfied. And obesity, preoperative pain duration lasting no less than 6 months, feel depression and symptom recurrence are connected with postoperative patients' dissatisfaction. While postoperative rehabilitation training for three months is a protective factor. Adequate preoperative and postoperative communication is necessary for patients with these risk factors.

## Data Availability

The original contributions presented in the study are included in the article/Supplementary Material, further inquiries can be directed to the corresponding author.
